# Comments on the position UEMS paper “Maintaining Human Rights and recovery principles when Coercive Practices are considered”

**DOI:** 10.1192/j.eurpsy.2023.156

**Published:** 2023-07-19

**Authors:** S. S. Ivezic

**Affiliations:** University psychiatric hospital Vrapčce, Zagreb, Croatia

## Abstract

**Abstract:**

Prof. dr. sc. Slađana Štrkalj Ivezić, psychiatrist, University psychiatric hospital Vrapče, Croatia

The aim is to present the importance of educating psychiatrists to acquire competencies relate to knowledge, attitudes and behavior in holistic understanding of patients, implementation of principles of recovery and respect for human rights and use of alternative interventions to coercion in order to eliminate or reduce coercive practice such as involuntary hospitalizations and coercive measures. All the necessary competences such as clinical assessment, skills to form therapeutical relationship and application of evidence base interventions that can prevent or significantly reduce the use of coercive measures: de-escalation; availability of a comfort room with sensory modulation; a trained response team; joint crisis intervention, advance directives and successful multimodal strategies will be presented including the elimination of potential environmental triggers of aggression in the hospital setting. The training and education of psychiatrists on human rights, recovery and alternatives to coercive practice can abolish or significantly reduce coercion.

**Disclosure of Interest:**

None Declared

